# The Truth about Sanatoria

**Published:** 1912-01-06

**Authors:** Marcus Paterson

**Affiliations:** Medical Superintendent Brompton Hospital Sanatorium, Frimley.


### THE TRUTH ABOUT SANATORIA.

### Three Essential Points to Success.

By MARCUS PATERSON, M.B., B.S., Medical Superintendent Brompton Hospital Sanatorium, Frimley.

Unkind things are often said about sanatoria. Why? Because public bodies and private persons have built and equipped small institutions of 20 to 30 beds, instead of building large institutions with 150 to 250 beds. In consequence of this mistake they can seldom obtain economy or efficiency. Every sanatorium must have fully trained heads of the various departments, and these heads can just as easily supervise a large as a small staff. With a sanatorium of 30 beds it is almost impossible to offer a salary sufficient to induce first-class men to undertake the work, with the result that too often medical men have taken these posts at small salaries, because they themselves have been broken down in health by tuberculosis.

Of course, it does not follow that a medical man who has had consumption will make a successful superintendent of a sanatorium. Sanatorium treatment, carried out on modern lines, is not fitted for anyone who is not in robust health. I do not wish to be misunderstood; if a man has had tuberculosis and has recovered, and he is considered a good man for the post, there can be nothing against his appointment. My point is that it is false economy to offer small salaries and run the risk of converting sanatoria into homes for invalid doctors.

The remedy is for the various districts to combine and build large institutions, and pay salaries to induce the best men from our medical schools to take up sanatorium work. The present system puts a premium on inefficiency. To obtain the best results, it is obvious that sanatoria should have their share of the best medical men.

